# Defining Dysphoric Milk Ejection Reflex: using Premenstrual Dysphoric Disorder as a framework for proposing preliminary diagnostic criteria

**DOI:** 10.3389/fpsyt.2025.1694844

**Published:** 2026-02-03

**Authors:** Megan Howard, Teni Davoudian, Nicole H. Cirino

**Affiliations:** Department of Obstetrics and Gynecology, Baylor College of Medicine, Houston, TX, United States

**Keywords:** breastfeeding, diagnostic criteria, dysphoric milk ejection reflex, lactation, postpartum, PMDD, postpartum depression

## Abstract

Dysphoric Milk Ejection Reflex (D-MER) is a distinct neurobiological condition characterized by negative alterations in mental state in response to milk letdown during lactation. Symptoms vary by patient and can include feelings of sadness, anxiety or agitation. Importantly, the symptoms are brief, typically lasting no more than 5 minutes. Prevalence has been found between 6 and 27% of lactating women, but studies show heterogeneity, due in part to inconsistent definition. D-MER is not currently classified in the Diagnostic and Statistical Manual of Mental Disorders (DSM) or the International Classification of Diseases (ICD), which presents a challenge for researchers of the condition. The pathophysiology of D-MER is not well understood, but may be mediated by hormonal changes. In an attempt to begin to formalize classification of this condition, the authors explore the association with another recently classified, hormonally mediated and time sensitive condition: premenstrual dysphoric disorder or PMDD. Like D-MER, PMDD is characterized by heterogeneous symptoms that occur on a predictable timeline. The recent addition of a formal diagnostic category into the DSM helped facilitate an expansion of research into etiology and treatment of the condition. This paper will explore a pathway to classification of D-MER based on the current research using the framework of the DSM-5 diagnostic criteria for PMDD. We will conclude by outlining future research priorities that will help to better define this condition and differentiate it from other causes of emotional distress during lactation.

## Introduction

Dysphoric milk ejection reflex (D-MER) is the momentary experience of negative emotions during milk letdown in lactation. D-MER was first described in case reports as an emotional “drop” that coincided with milk letdown (1, 2). These early reports noted a variety of emotions experienced, broadly categorized into “depression,” “anxiety” and “anger” (2). These symptoms are notable for their frequency and brevity: most patients report that they occur with the majority of lactation episodes and correspond specifically to milk letdown, resolving in under 5 minutes (3).

Despite the brevity of symptoms, D-MER has significant impacts on both breastfeeding and mental health outcomes. Many patients discontinue breastfeeding (4), resulting in feelings of guilt, isolation, shame, and inadequacy as a mother (5). D-MER has also been linked with lower breastfeeding self-efficacy, increased difficulty with bonding, and higher depression (4, 6, 7). Nearly 30% of patients reporting D-MER symptoms experienced thoughts of hurting themselves or others directly connected with milk letdown (3), and D-MER patients are twice as likely to report thoughts of self-harm than those without (4). In contrast, typical experiences during milk letdown include positive emotional responses, reduced stress, increased bonding, and the feeling of calmness (8).

There has been a growing awareness of D-MER in the psychiatric literature, and a call for further study and clear definition (9, 10), as it is not currently included in the Diagnostic and Statistical Manual for Mental Disorders (DSM). There is an awareness of the importance of mental health conditions in the management of breastfeeding and lactation (11), however, D-MER is not formally recognized with a clinical protocol from the Academy of Breastfeeding Medicine (12), nor included as relevant content for certification through the International Board of Lactation Consultant Examiners (13). Thus, women with this condition to go undiagnosed and untreated whether they present to psychiatry or lactation professionals. Additionally, the lack of standardized definition hinders efforts to better understand the condition through rigorous research.

The goal of this paper is to describe D-MER from a thorough review of the literature, to discuss the rationale for including D-MER as a mental disorder, and to propose a preliminary definition for the condition. Because the timing of D-MER symptoms is the key defining criteria, we based the diagnostic criteria on those for premenstrual dysphoric disorder (PMDD), another condition defined by specific timing of symptoms. While research into D-MER remains preliminary, this definition will be a step forward to promote clinical recognition and systematic scientific inquiry into this condition.

## Approach

A thorough review of the literature was conducted to ensure completeness of data regarding D-MER. Medline and Psychinfo were searched using a search strategy developed in consultation with a medical librarian. Because the phrase “dysphoric milk ejection reflex” may not be universally recognized, the terms dysphoria, dislike or disgust were included, and any papers that used these terms within 5 words of various lactation terms in the title, abstract or key words were included. Search was limited to 2010 or later and to studies conducted in humans. A total of 41 articles were retrieved after elimination of duplicates. Titles and abstracts were examined for relevance, and 12 were determined to be not relevant. Six references were focused specifically on Breastfeeding Aversion Response (BAR), and these were reviewed separately. The remaining 29 articles were reviewed in full text and included.

## Review of D-MER literature

### Timing of D-MER symptoms

**Figure 1:**
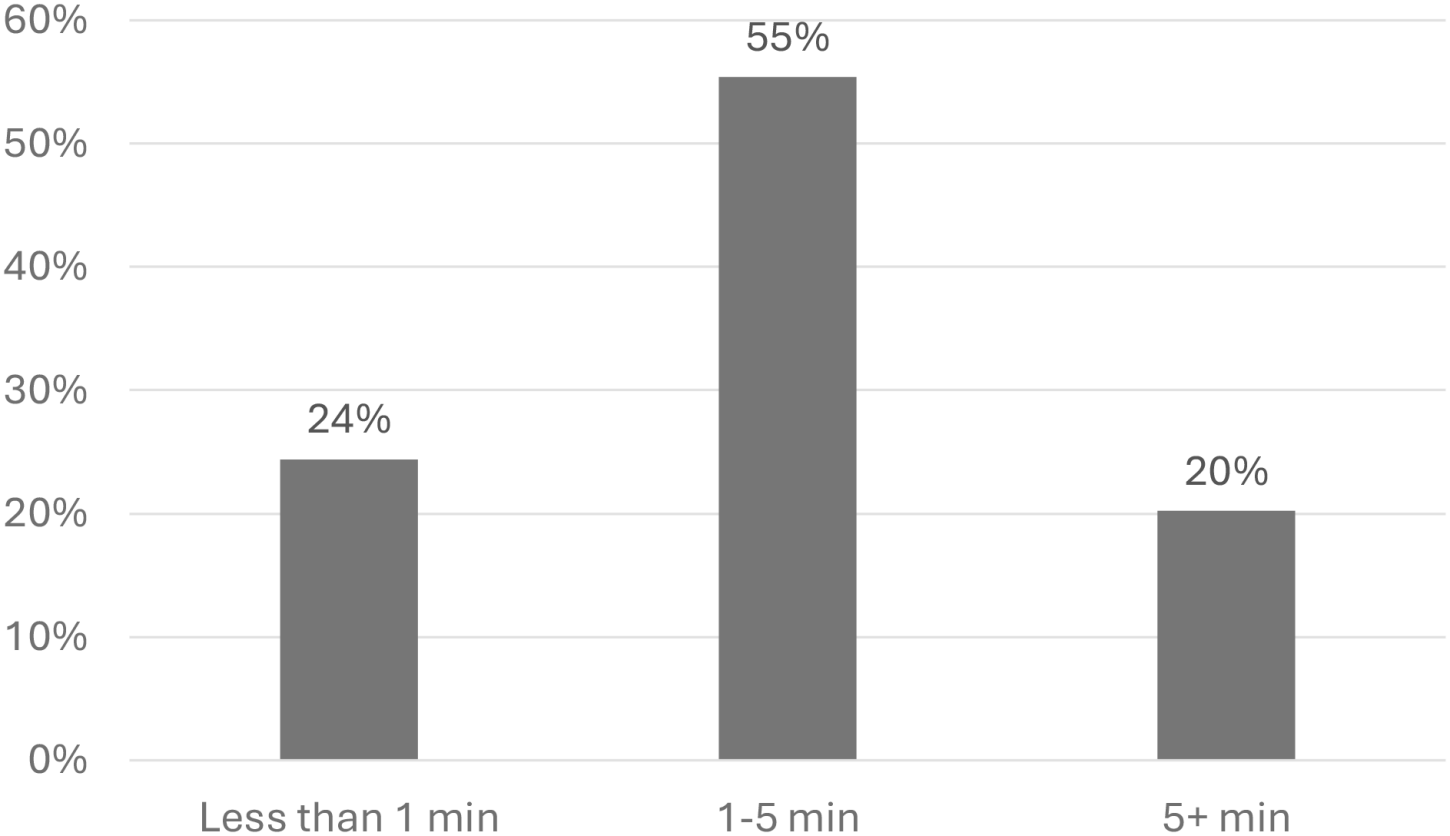
Pooled data regarding duration of D-MER symptoms reported: represents pooled data from five studies (3, 4, 6, 7, 14) for a total n of 381 patients.

The specific timing of D-MER symptoms sets it apart as a distinct clinical entity; symptoms are characteristically brief and correspond with milk letdown. Pooling the data of five studies reporting duration of D-MER symptoms shows that approximately 24% of respondents indicate that their symptoms last less than one minute, and an additional 55% report resolution in one to five minutes (**Figure 1**) (3, 4, 6, 7, 14).

**Figure 2:**
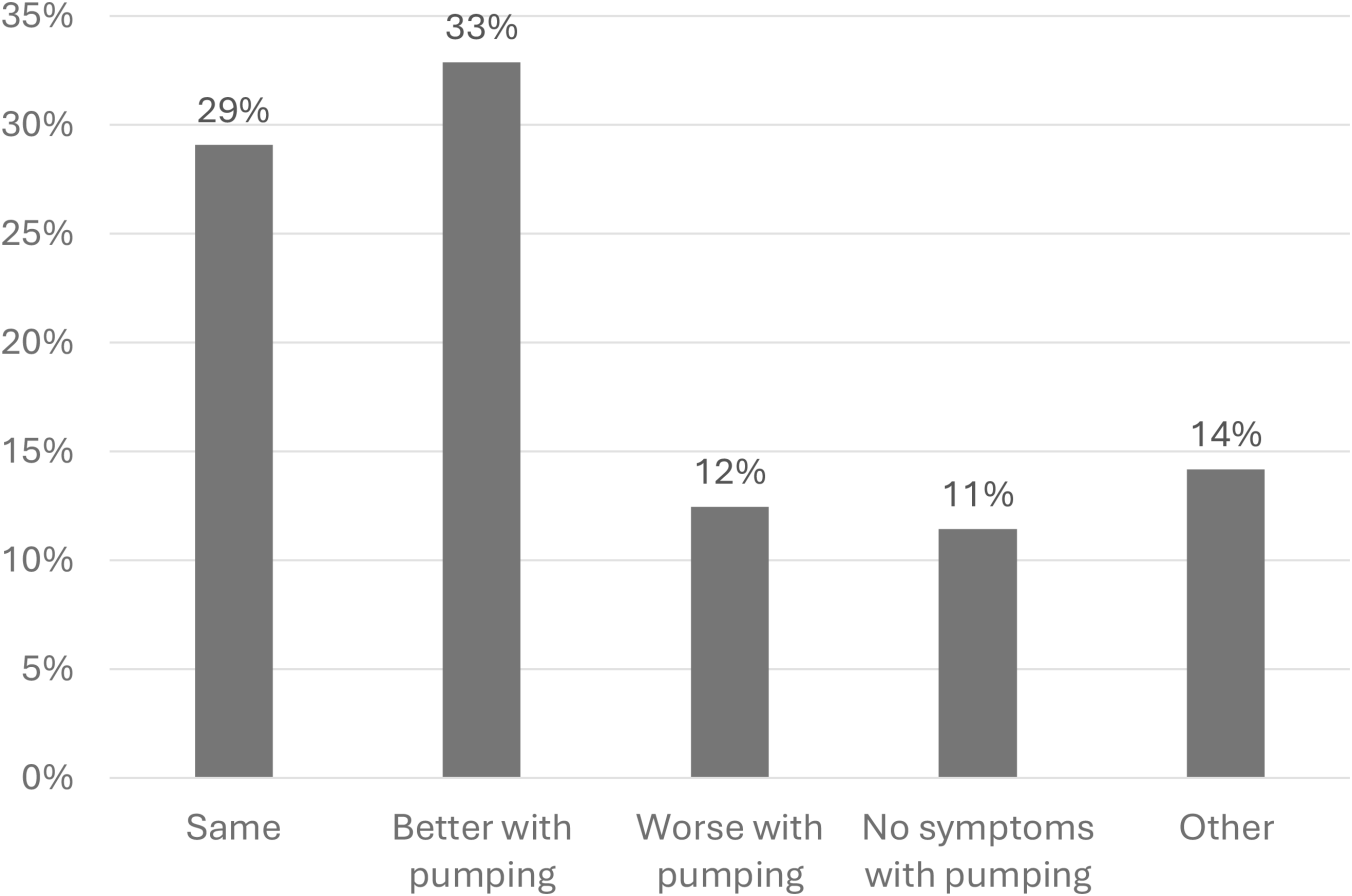
Pooled data regarding D-MER symptoms in response to breastfeeding vs pumping: Symptom change with direct breastfeeding as compared to milk expression using a breast pump. Represents pooled data from four studies (3, 6, 7, 14) for a total n of 289 patients.

Letdown, also known as the “milk ejection reflex” is a physiological process mediated by oxytocin, which is released from the posterior pituitary, triggering the contraction of myoepithelial cells, releasing milk from alveoli into milk ducts (15). It is triggered most commonly by infant suckling or mechanical stimulation of the nipple by a breast pump, though it can occur spontaneously or be triggered by visual or emotional stimuli. Multiple case reports have described women with D-MER symptoms that are triggered by spontaneous letdowns as well as breastfeeding and pumping (2, 16). Studies which have asked women to compare symptoms elicited by breastfeeding to those elicited by pumping show that approximately half of respondents find their symptoms are less intense with mechanical expression of milk rather than direct breastfeeding (**Figure 2**) (**3**, 6, 7, 15), although it is unclear why this may be.

The specificity of the timing of D-MER and its relationship to letdown suggests a role for oxytocin as a mediator (17), though this is yet to be definitively established through biological studies. Other proposed mechanisms include momentary drops in dopamine (2) or involvement of vasopressin, an anxiogenic hormone with a reciprocal relationship with oxytocin which tends to predominate in situations of adversity (18, 19).

The onset and progression of D-MER symptoms through the postpartum are variable but often begin soon after delivery. Most women report symptom onset within the first month postpartum (6), and in one study nearly 40% of mothers reported symptom onset with the first feeding (4). Symptoms tend to improve or even resolve with time for many women (6, 14). Duration of symptoms varies, though in all reports, symptoms resolve with weaning (1, 2, 16, 20).

### Differential diagnosis

D-MER is frequently misdiagnosed as postpartum depression (PPD) (2, 21). PPD is classified by the DSM as a Major Depressive Episode with perinatal onset (onset during pregnancy or within 4 weeks after delivery) (22). Symptoms occur for most of the day, more days than not for a minimum of two weeks (22). While there is significant overlap in the symptoms experienced in PPD and D-MER, the timing of the symptoms is different, as most women with D-MER report feeling happy between letdowns (3).

Another condition that is sometimes confused for D-MER is breastfeeding aversion response (BAR), which is characterized by feelings of agitation and disgust while breastfeeding, often associated with the urge to unlatch or a sensation of the skin crawling (23, 24). The symptoms of BAR occur throughout the time that the baby is latched, and have not been described in pumping or spontaneous letdowns. This is distinct from D-MER where symptoms are limited to the time around letdown, and can occur with spontaneous letdowns as well as pumping (2, 20, 21).

### Epidemiology

Little is currently known about D-MER risk factors. Several studies have shown a correlation between D-MER and any pre-pregnancy psychiatric diagnosis (4, 25). Correlations have also been found with current postpartum depression (25), panic attacks (26), and “another mood disorder” (not depression or anxiety) (7). One cross-sectional study of lactating women found that factors such as higher education and immigration status also correlated with elevated D-MER risk (25).

The prevalence of D-MER has been reported in the literature between 6-27% of lactating parents (**Table 1**) (3, 4, 6, 7, 14, 26–28). These studies have been performed in widely disparate patient populations, and the timing postpartum ranges from 2 days to 3 years. There is also no consensus definition to identify D-MER cases. Not surprisingly, those studies with more stringent criteria for identifying D-MER report lower prevalence. For instance, the two studies that required D-MER symptoms to occur most or all of the time had the two lowest prevalence rates at 6% and 5.9% (4, 7). This highlights the need for a standardized definition of D-MER.

**Table 1:** Summary table for D-MER prevalence studies.

Study	Symptom description	Symptom timing	N	Timing postpartum	How collected	Prevalence	
Ureno 2019 (3)	“negative emotional response”	“during MER”	164	6–8 weeks	Chart review from screening question at postpartum visit	9.1%
Nguyen 2024 (7)	“dysphoria”	“after latching but prior to milk letdown” and “during milk letdown” *Also required symptoms occur “most of the time” or “always”*	201	4–12 weeks	Online survey	6%
Moriyama 2024 (27)	“unpleasant symptoms such as anxiety, sadness, irritability and panic are experienced while breastfeeding”	“temporary unpleasant symptoms occur reflexively at the milk ejection”	202	3 years	Paper questionnaires at child’s 3-year checkup	15.4% for child at 3-year visit; 23.3% of mothers reported with this or other child
Kacir 2024 (6)	“negative emotions that you cannot control, such as depression, anxiety or anger	“before breastfeeding/just before you start breastfeeding” Required “yes or “sometimes”	141	0–2 years	Online questionnaire through social media for women experiencing “unpleasant emotions while breastfeeding”	27.7%
Solmonovich 2024 (28)	List of 21 symptoms from “very mild” to “severe”	“sudden and temporary” “Immediately before milk letdown”	78	1–2 days	In person during delivery admission; education given about D-MER and patients asked to participate in daily surveys	26.9%
Zutic 2024 (4)	“unpleasant emotions”	“right before the start and in the first few moments of milk release” *Required “often” or “almost always”*	711	Up to 12 months	Online through Facebook and personal connections	5.9%
Howard 2025 (26)	“negative emotions such as anxiety, unpleasantness or dread”	“during milk letdown”	271	0–75 weeks, avg 11.5 weeks	Chart review from intake packet at breastfeeding medicine clinic	15.5%
Cappenberg 2025 (14)	“D-MER is and abrupt, often overwhelming wave of negative emotions that come only during milk-letdown”	“wave of negative emotions that come during milk letdown”	1469	0–18 months	Online survey in Germany	14.2%

## Working toward a D-MER diagnosis

Proposal of diagnostic criteria for D-MER implies its existence as a distinct diagnostic entity. D-MER is a disturbance in emotion regulation, associated with significant distress and frequently resulting in premature weaning, consistent with the DSM definition of a mental disorder (22). Robins and Guze postulated a 5-phase approach to defining a disorder in their seminal 1970 paper (29). These phases included 1. Clinical description, 2. Laboratory studies, 3. Delimitation from other disorders, 4. Long term studies, and 5. Family studies. They additionally note that progression through the phases is neither linear nor chronological, and that the five phases interact with one another. The literature on D-MER has thus far focused on clinical description of the condition, with a focus on the quality of symptoms. Laboratory studies or biomarker studies in clinical samples have been lacking, and these studies are needed to move the field forward. However, clearly defining D-MER is a prerequisite for further biological studies, because without a consensus definition, study of biomarkers is unlikely to be meaningful. We propose these criteria in an effort to distinguish D-MER from other disorders, with the awareness that much research remains to be done in order to develop them more fully.

The hallmark of D-MER is the specific timing of the symptoms in relation to letdown. Another condition with timing of symptoms as a characteristic feature is PMDD. This paper uses the diagnostic criteria for PMDD as a framework from which we have developed preliminary diagnostic criteria for D-MER. Both PMDD and D-MER are linked to fluctuations in female sex hormones, however there is not currently evidence to suggest a correlation in these two conditions. Rather, we have drawn upon the structure of the DSM criteria for PMDD because of the emphasis in those criteria on the very specific timing of the symptoms. Both conditions also share cyclical, transient patterns of symptom manifestations with symptoms minimal or absent at other times (3, 22). Importantly, both conditions include heterogeneous affective symptoms that may include sadness, anxiety or irritability as well as somatic symptoms, such as bloating for PMDD and nausea or hollowness in the stomach for D-MER (3, 30).

## Premenstrual dysphoric disorder

Currently, the Diagnostic and Statistical Manual of Mental Disorders 5 (DSM-5) defines Premenstrual Dysphoric Disorder (PMDD) by the presence of at least five symptoms that occur specifically in the week before menses and become minimal or absent after menses (22). Symptoms must include one or more core symptoms plus one or more additional symptoms for a total of at least five symptoms (**Table 2**). These symptoms must result in clinically significant distress or functional impairment, must not be better explained as an exacerbation of another psychiatric disorder, and must be confirmed by prospective daily ratings.

**Table 2:** Proposed diagnostic criteria for dysphoric milk ejection reflex.

	PMDD	D-MER	Areas for further research
Timing of symptoms	A) In the majority of menstrual cycles, at least 5 symptoms must be present in the final week before the onset of menses, start to improve within a few days after the onset of menses, and become minimal or absent in the week post menses	A. One or more symptom must be present during the moments before and/or during milk letdown, improve within 5 minutes of onset, and become minimal or absent between letdowns. Symptoms may recur with subsequent letdowns during a single lactation episode or spontaneous letdowns not associated with a lactation episode	Define frequency and duration of symptoms
Core Symptoms	B) One or more of the following symptoms must be present: 1) Marked affective lability (e.g., mood swings, feeling suddenly sad or tearful, or increased sensitivity to rejection) 2) Marked irritability or anger or increased interpersonal conflicts 3) Markedly depressed mood, feelings of hopelessness, or self-deprecating thoughts 4) Marked anxiety, tension, and/or feelings of being keyed up or on edge	B. One or more of the following symptoms must be present: 1. Marked anxiety or panic, including feelings of tension or dread 2. Markedly depressed mood including feelings of sadness, hopelessness, tearfulness or worthlessness 3. Marked irritability, agitation or anger	Cognitive symptoms, such as suicidal ideation
Additional Symptoms	C) One (or more) of the following symptoms must additionally be present to reach a total of 5 symptoms when combined with symptoms from criterion B above 1) Decreased interest in usual activities 2) Subjective difficulty in concentration 3) Lethargy, easy fatigability, or marked lack of energy 4) Marked change in appetite; overeating or specific food cravings 5) Hypersomnia or insomnia 6) A sense of being overwhelmed or out of control 7) Physical symptoms such as breast tenderness or swelling; joint or muscle pain, a sensation of “bloating” or weight gain	C. In addition, somatic symptoms may be present but are not adequate to make a diagnosis of D-MER: 1. Nausea, churning or hollow stomach 2. Pain	Additional somatic symptoms including panic symptoms like shortness of breath, racing heart, sweating
Severity/ Impairment	D) The symptoms are associated with clinically significant distress or interference with work, school, usual social activities, or relationships with others.	D. The symptoms are associated with clinically significant distress or functional impairment, including diminished breastfeeding satisfaction or impairment in bonding with infant	Further explore impact on distress and interaction with other psychiatric illnesses Explore impact on breastfeeding, bonding and other relationships
Consider Other Psychiatric Disorders and medical causes for symptoms	E) The disturbance is not merely an exacerbation of the symptoms of another disorder, such as major depressive disorder, panic disorder, persistent depressive disorder (dysthymia) or a personality disorder (although it may co-occur with any of these disorders). G) The symptoms are not attributable to the physiological effects of a substance (e.g., drug abuse, medication or other treatment) or another medical condition (e.g., hyperthyroidism).	E. The disturbance is not merely explained by the symptoms of another disorder such as depression, panic, obsessions, phobias or anxiety F. The disturbance is not based solely on pain or physical discomfort associated with breastfeeding	Explore relationship with other psychiatric conditions such as postpartum depression Explore relationship with pain
Confirmation of the disorder	F) Criterion A should be confirmed by prospective daily ratings during at least 2 symptomatic cycles (although a provisional diagnosis may be made prior to this confirmation)	*Currently no data to suggest prospective daily ratings needed, but further research is needed*	Determine usefulness of prospective daily ratings, establish timing (frequency, duration), and relationship with distress/impairment

The symptoms of PMDD are understood to be the result of an abnormal sensitivity in the brain to normal hormonal fluctuations, particularly estradiol and progesterone (30). Neuroimaging and biomarker studies suggest that individuals with PMDD have altered GABAergic and serotonergic signaling, especially in the amygdala and prefrontal cortex, which are involved in mood regulation (31). These changes are not due to abnormal hormone levels, but rather to differential neurobiological responses to the hormonal fluctuations which occur in the luteal phase of the menstrual cycle. While similar hormonal fluctuations occur in all naturally cycling women, those with an underlying predisposition may become symptomatic.

## Diagnosis of hormonally-mediated conditions and the DSM

It has been only in the past 30 years that the DSM began to recognize conditions that are precipitated by reproductive events. The DSM-IV, published in 1994, was the first version to include the "postpartum onset" specifier for major depressive disorder, updated in DSM-5 to the “peripartum onset” specifier. Premenstrual disorders, described in medical literature as early as the 4^th^ century BC, were first included in the appendix of DSM-III (32). The prospect of moving PMDD from DSM-III’s appendix to DSM-IV’s main text in 1993 was met with opposition and controversy and ultimately, the DSM-IV task force decided to maintain PMDD in the appendix, despite agreeing upon PMDD symptomatology, course of symptoms and the potential benefits of treatment. PMDD was transitioned from DSM-IV’s appendix to DSM-5’s main text in 2014 (22).

Six years prior to DSM-5’s recognition of PMDD as a standalone diagnosis, the International Society for Premenstrual Disorders provided a unified approach to diagnosing premenstrual disorders in hopes of informing discussion for the next editions of ICD and DSM (33). In 2012, The Mood Disorders Work Group for DSM-5, which was comprised of representatives from various countries, evaluated previous diagnostic criteria for PMDD and found the criteria to be aligned with additional data that had become available (34). There were several other factors that supported this smooth transition including United States Food and Drug Administration’s recognition of PMDD as a distinct disorder and research findings showing consistent prevalence rates of PMDD across countries (35). Following DSM-IV’s recognition of PMDD, the magnitude of scientific evidence on PMDD has burgeoned and individuals afflicted with this condition can now receive research-informed care (32).

While research into D-MER is still in early stages, establishing preliminary diagnostic criteria will allow this condition to be reliably distinguished from other conditions, facilitating further clinical and scientific progress. As with PMDD, we anticipate that further study will result in the modification of these criteria to continue to reflect the state of science and to promote further diagnostic validity of this condition.

## Proposal of diagnostic criteria for D-MER

Proposed diagnostic criteria for D-MER are listed in **Table 2**. Criterion A for the diagnosis of PMDD defines the timing as occurring primarily in the week before menses, improving after the onset of menses and becoming minimal or absent in the follicular phase of the menstrual cycle. For D-MER, we propose defining the timing to occur in the moments before and/or during milk letdown and improving within five minutes, becoming minimal or absent between letdowns as has been consistently described in the literature (2, 3, 20, 21). PMDD is also defined as occurring in the majority of menstrual cycles. For D-MER, we do not yet have clear evidence that most letdowns must be symptomatic for the syndrome to impact a patient’s life.

Criterion B for the diagnosis of PMDD defines the core symptoms of affective lability, irritability/anger, depressed mood, and anxiety/tension. The DSM notes that one or more must be present, with a total of 5 symptoms which include at least one from the additional symptoms listed in criteria C. It is noteworthy that three of the four symptom categories described in the diagnostic criteria for PMDD correspond to three symptoms categories originally described for D-MER: depression, anxiety and anger (2). These categories have continued to capture additional D-MER symptoms described, and have been used in various studies to categorize symptoms (6, 20).

Criterion C describes additional symptoms, including a number of somatic symptoms such as appetite changes, breast tenderness and bloating. Different physical symptoms have been described for D-MER including breast pain and a sensation of nausea or churning/hollowness in the stomach (3, 26). One important area for future research is to further explore the contribution of breast/nipple pain to D-MER symptoms.

Criterion D for PMDD requires clinically significant distress or impairment to functioning. The evidence thus far supports an association with D-MER symptoms and impaired bonding, breastfeeding and mental health outcomes (4), however it is possible that this is a bidirectional relationship and merits a more nuanced examination. To define distress and impairment for D-MER, we have included diminished breastfeeding satisfaction or impairment in bonding, though further research may reveal other outcomes that are impacted by D-MER. Further research is also needed to define how D-MER may impact relationships other than that between the breastfeeding dyad.

Criteria E and G for PMDD both involve ruling out other medical or psychiatric conditions that may cause similar symptoms. Our proposed criterion E for D-MER emphasizes that symptoms should not be caused primarily by other mental health conditions including depression and anxiety. Importantly, these disorders may co-occur with D-MER, however the D-MER symptoms are defined by an abrupt change to a patient’s baseline mental state. Criterion F also highlights that the mood disturbance is not based solely on physical discomfort or pain associated with breastfeeding.

Finally, Criterion F for PMDD describes confirmation of the disorder by prospective daily rating during at least 2 symptomatic cycles. Currently, there are no studies that have examined prospective ratings of D-MER symptoms. More research is needed to determine if such ratings are useful in the diagnosis of D-MER and to determine cutoffs for frequency and duration of symptoms.

## Discussion

There is clear precedent in both DSM and ICD systems that reproductive life events that lead to alterations of sex hormones can precipitate distinct psychiatric conditions characterized by heterogeneous symptoms that occur with specific timing relative to hormonal changes. The evolution of PMDD as a distinct diagnostic category and the addition of peripartum specifiers in diagnostic systems has facilitated standardization in research and allowed clinicians to recognize and treat these clinically impactful psychiatric conditions.

Existing data regarding D-MER supports it as a distinct psychiatric condition given its specific onset, phenotype, phenomenology and prognosis. Familiarity and genetic vulnerability have yet to be established, similar to PMDD prior to its inclusion in the DSM. The current lack of standardized diagnostic criteria for D-MER presents a major limitation to identification of D-MER cases in clinical and research settings. Thus, clearly delineating this condition is a critical step in legitimizing and advancing scientific inquiry, allowing for development of effective interventions, investigation of biomarkers and identification of risk factors.

While the DSM diagnostic criteria are widely used for both clinical and research purposes, the level of evidence currently available for D-MER is not at a point where it is reasonable to propose inclusion in the DSM. The criteria proposed are intended to be specific enough for both research and clinical use and flexible enough to incorporate additional information as it accumulates. As research into D-MER progresses, these criteria may be adapted into DSM or ICD frameworks. As noted by Robins and Guze (1970), “the process [of establishing diagnostic validity] is one of continuing self-rectification and increasing refinement” (29).

We propose that next steps in research on D-MER focus on further characterization of the condition to support the ongoing development of an evidence-based definition, including exploration of additional cognitive and somatic symptoms; the utility of prospective daily ratings; and the impact of D-MER on important breastfeeding and maternal mental health outcomes. It is our hope that these criteria will provide an important step forward in defining D-MER to facilitate the research needed to better characterize the disorder, establish evidence-based treatment, and ultimately to improve clinical outcomes.

## Data Availability

The original contributions presented in the study are included in the article/supplementary material. Further inquiries can be directed to the corresponding author.
